# Genome-Wide Association Screening and Candidate SNP Analysis Identify an Intergenic Variant Near *CTNND*2 Associated with Litter Size Traits in Saanen Dairy Goats

**DOI:** 10.3390/vetsci13090961

**Published:** 2026-09-14

**Authors:** Jianqing Zhao, Shuhao Guo, Jianwu Li, Wenjie Sun, Yuqi Ma, Wei Wang, Weiming Gao

**Affiliations:** 1 College of Animal Science, Xinjiang Agricultural University, Urumqi 830052, China; zhao2021@nwafu.edu.cn (J.Z.);; 2 Shaanxi Key Laboratory of Molecular Biology for Agriculture, College of Animal Science and Technology, Northwest A&F University, Yangling 712100, China; 3 Xinjiang Uygur Autonomous Region Academy of Animal Sciences, Urumqi 830011, China

**Keywords:** Saanen dairy goat, intergenic SNP, *CTNND*2, genome-wide association study, litter size, live-born kids, candidate marker

## Abstract

Litter size is an economically important reproductive trait in dairy goats, but its genetic improvement remains challenging because of its complex genetic architecture and the influence of multiple environmental factors. This study aimed to identify genomic loci associated with litter size-related traits and to evaluate the potential of an intergenic variant near the candidate gene *CTNND*2 as a molecular marker in Saanen dairy goats. A total of 341 does with reproductive records were analyzed using a genome-wide association study (GWAS), followed by candidate SNP genotyping and association analysis. A shared association signal for litter size and live-born kid number was identified on chromosome 20, leading to the selection of the intergenic SNP CM048886.1:g.61357858G>A located near *CTNND*2 for further evaluation. Three genotypes (GG, AG, and AA) were detected, and the locus exhibited moderate genetic diversity (PIC = 0.27) while conforming to Hardy–Weinberg equilibrium. Association analysis revealed nominal associations between the SNP and both litter size (*p* = 0.0445) and live-born kid number (*p* = 0.0448), whereas no significant association was observed with average kid birth weight (*p* = 0.125). Goats carrying the A allele showed numerically higher litter size and live-born kid number than those carrying the G allele. These findings identify CM048886.1:g.61357858G>A, an intergenic variant near *CTNND*2, as a promising candidate marker for litter size-related traits in Saanen dairy goats and provide a foundation for future validation studies and the development of molecular breeding strategies to improve reproductive performance.

## Simple Summary

Litter size is an important determinant of reproductive efficiency and economic profitability in dairy goat production, but improving this trait through conventional breeding is challenging because it is influenced by multiple genes and environmental factors. In this study, we identified a chromosome 20 genomic region associated with litter size-related traits in Saanen dairy goats and detected an intergenic variant near the candidate gene *CTNND*2 (CM048886.1:g.61357858G>A) that was associated with favorable litter size and live-born kid number. No association was observed with average kid birth weight. These findings suggest that this variant may represent a promising candidate marker for improving reproductive performance in dairy goats and provide a foundation for future validation studies and the development of marker-assisted breeding strategies.

## 1. Introduction

Dairy goats are important livestock for milk production, rural economies, and diversified animal-protein supply. Global dairy goat production has expanded because goat milk and related products contribute to human nutrition and farm income [1]. Saanen goats are widely used in commercial dairy-goat production because of their high milk yield, adaptability, and value in structured breeding programs [1,2]. In addition to milk production, reproductive efficiency is a key determinant of herd expansion, replacement rate, and overall economic performance; therefore, genetic studies of reproductive traits are relevant to both production efficiency and long-term breeding design [2].

Litter size directly affects kid output, replacement capacity, and the efficiency of genetic improvement. However, litter size is a complex quantitative trait with low-to-moderate heritability and substantial environmental sensitivity. Published estimates for litter-size traits in goats vary markedly among breeds, parity structures, and statistical models, ranging from very low values close to zero or 0.006–0.074 in some indigenous and Markhoz goat populations to approximately 0.11 in Creole goats and up to about 0.37 in parity-specific estimates from Spanish dairy goat breeds [3,4,5,6]. Phenotype-based selection is therefore slow, particularly because reproductive records are collected only after sexual maturity and parturition. Genome-wide association studies (GWAS) and candidate-gene analyses provide complementary approaches for identifying genomic markers associated with complex reproductive traits [2,3,7,8].

Recent goat GWAS have moved beyond single-gene association testing and have identified population-specific loci for reproductive traits. In Markhoz goats, GWAS of litter size at birth and weaning identified significant SNPs on chromosomes 2, 20, and 21 and candidate genes including *GABRA*5, *AKAP*13, *SV*2*B*, *PPP*1*R*1*C*, *SSFA*2, and TRNAS-GCU [3]. In Dazu Black goats, whole-genome resequencing of reproductive and udder traits in 150 animals implicated candidate genes such as *SLC*9*A*8, *GLRB*, *GRIA*2, *GASK*1*B*, and other annotated loci for litter size [7]. In Youzhou Dark goats, analyses based on genome-wide SNP markers in 206 goats identified a litter-size-associated SNP on chromosome 11 and additional reproductive candidate genes highlighted through selection-signature and runs-of-homozygosity analyses, including *TSHR*, *ANGPT*4, *CENPF*, *CHST*1, *PIBF*1, *DACH*1, *DIS*3, *COL*4*A*1, *PRKD*1, and *DNMT*3*B* [8]. These studies demonstrate that prolificacy is genetically heterogeneous across breeds and that marker effects require population-specific validation before breeding use.

*CTNND*2 encodes delta-catenin, a protein involved in cadherin-mediated cell adhesion and Wnt/β-catenin signaling, which plays an important role in ovarian follicular development and granulosa cell function [9,10,11,12,13]. These biological functions suggest that the genomic region surrounding *CTNND*2 may harbor genetic variants influencing reproductive traits. However, the potential contribution of this region to litter size in dairy goats has not been investigated.

Therefore, this study aimed to identify genomic regions associated with litter size-related traits in Saanen dairy goats through genome-wide association analysis and to evaluate the association between the intergenic SNP CM048886.1:g.61357858G>A, located near *CTNND*2, and reproductive performance. The findings are expected to improve our understanding of the genetic architecture of litter size and provide candidate molecular markers for future dairy goat breeding.

## 2. Materials and Methods

### 2.1. Ethics Statement, Animals, and Phenotypic Records

The study was approved by the Institutional Animal Care and Use Committee of Northwest A&F University (approval number: 24-522; date of approval: 10 September 2024). All applicable institutional and national guidelines for the care and welfare of animals were followed during sampling and phenotypic recording.

A total of 341 healthy Saanen dairy goat does with complete individual-level reproductive records were included in this study. The animals were maintained in a single herd under a standardized management system at the experimental station. Does were housed under the same general rearing conditions, fed according to physiological stage using the farm routine diet, and had free access to clean water. Vaccination, health care, and breeding management were applied uniformly within the herd. The does were 1–5 years old at kidding, and the parity of the recorded kidding event ranged from 1 to 4. All does were bred by artificial insemination. Reproductive records were collected from annual kidding records and, to reduce seasonal heterogeneity, were restricted to spring kidding records.

Each doe contributed one phenotypic record to the association analysis; therefore, repeated kidding records from the same doe were not treated as independent observations. For each doe, litter size (T1) was defined as the total number of kids born in the recorded kidding event, live-born kid number (T2) was defined as the number of live-born kids in the same kidding event, and average kid birth weight (T3) was defined as the average birth weight of kids born in that kidding event. Blood samples (5 mL) were collected from the jugular vein into EDTA anticoagulant tubes and stored at −80 °C until DNA extraction. Phenotypic records exceeding three standard deviations from the trait mean were excluded before association analyses. Age at kidding and parity of the recorded kidding event were retained as covariates for association modeling.

### 2.2. Genome-Wide SNP Dataset, Quality Control, and SNP Distribution

Genome-wide SNP information was used for preliminary association screening. The genome-wide dataset was generated by whole-genome resequencing following the workflow described in our previous dairy-goat genomic study [14]. Briefly, paired-end sequencing libraries with an average insert size of approximately 500 bp were constructed and sequenced on the Huada T7 platform, with an average read length of 150 bp. Raw paired-end reads were filtered using Trimmomatic v0.39. Clean reads were aligned to the goat reference genome (ARS1.2/GCF_001704415.2) using BWA-MEM v0.7.15, and SAMtools was used for BAM indexing and processing. The BAM files were sorted, and potential duplicate reads were removed using Picard. SNP calling was performed using the HaplotypeCaller, GenotypeGVCFs, and SelectVariants modules in GATK v3.8. Variant filtration was conducted using the VariantFiltration module with the following criteria: QD < 2.0, FS > 60.0, MQ < 40.0, MQRankSum < −12.5, ReadPosRankSum < −8.0, SOR > 3.0, and abnormal sequencing depth outside the range of one-third to three times the average depth.

For the present GWAS, 341 Saanen dairy goat does with complete genotype and reproductive phenotype records were retained. Variant quality control was further conducted using PLINK v1.9 [15]. Individuals with a missing genotype rate greater than 0.10 were excluded, and SNPs with a minor allele frequency lower than 0.05 were removed. Non-autosomal and duplicate markers were also excluded. After quality control, 341 individuals and 14,597,388 autosomal SNPs were retained for association analysis. SNP density was calculated in 1 Mb windows across the 29 autosomes to evaluate genome-wide marker coverage.

### 2.3. Population Structure and Genome-Wide Association Analysis

Population structure and relatedness were evaluated using principal component analysis (PCA) [16] and a genomic kinship matrix. PCA was performed to inspect whether the Saanen dairy goat population contained major subgroups that could confound association testing. The genomic kinship matrix was included in the mixed model to account for relatedness among individuals. Thus, PCA was used mainly as a diagnostic assessment of population structure, whereas the kinship matrix was incorporated directly into the GWAS model.

GWAS for litter size, live-born kid number, and average kid birth weight were performed using GEMMA v0.98.5 under a linear mixed-model framework [17,18]. The model was written as:

*y* = *Wα* + *xβ* + *u* + *e*,

where y is the vector of individual-doe phenotypic values; W is the covariate matrix containing the intercept, age at kidding, and parity of the recorded kidding event; *α* is the vector of covariate effects; x is the genotype vector for each SNP marker; *β* is the marker effect; u is the random polygenic effect with covariance proportional to the genomic kinship matrix; and e is the residual error. Genome-wide significance was determined based on the number of quality-controlled SNPs. The genome-wide significance threshold was corrected by the Bonferroni test (0.05/14,597,388). Manhattan plots and Q-Q plots were drawn using the qqman package in R 4.6.1 [19].

### 2.4. Candidate SNP Selection, Primer Design, and PCR Amplification

Based on the GWAS results, a shared chromosome 20 association interval for litter size and live-born kid number was selected for candidate-SNP analysis. The candidate interval was delimited as CHR20 on ARS1.2/GCF_001704415.2. CM048886.1:g.61357858G>A is an intergenic SNP located within this candidate interval and approximately 552,627 bp from *CTNND2*. The SNP was selected because it was located in the shared chromosome 20 signal region, was close to a biologically plausible candidate gene, and could be reliably amplified for Sanger sequencing. This selection strategy does not establish *CTNND2* as the causal gene. Primers were designed according to the chromosome 20 sequence of the goat reference genome, and the expected amplicon length was 659 bp (Table 1).

Genomic DNA was extracted from blood samples collected from the 304 Saanen dairy goats included in the candidate SNP validation analysis using a commercial blood genomic DNA extraction kit according to the manufacturer’s instructions. DNA concentration and purity were assessed using a NanoDrop 1000 spectrophotometer (Thermo Fisher Scientific, Waltham, MA, USA), and DNA integrity was checked by 1% agarose gel electrophoresis. PCR amplification was performed in a 20 μL reaction containing 1 μL forward primer (1 μmol/L), 1 μL reverse primer (1 μmol/L), 1 μL genomic DNA template, 0.2 μL PrimeSTAR Max DNA polymerase, 2 μL dNTPs, 10 μL 2× GC buffer II, and 4.8 μL nuclease-free water. The cycling conditions were 95 °C for 3 min; 34 cycles of 94 °C for 15 s, 60 °C for 15 s, and 72 °C for 45 s; and final extension at 72 °C for 10 min. PCR products were examined by 1% agarose gel electrophoresis and submitted for Sanger sequencing.

### 2.5. Genotyping, Genetic Diversity, and Candidate SNP Association Analysis

Genotypes were determined by visual inspection of Sanger sequencing chromatograms using Chromas v2.6.5 software. Allele and genotype frequencies were calculated by direct counting. Observed heterozygosity (Ho), expected heterozygosity (He), effective allele number (Ne), and polymorphism information content (PIC) were calculated to evaluate genetic diversity [20,21]. Deviation from Hardy–Weinberg equilibrium was tested using the chi-square test. The genotype calls obtained by PCR/Sanger sequencing were compared sample-by-sample with the corresponding genotype calls in the GWAS dataset for the same individuals. Complete concordance between the two genotyping methods supported technical genotype confirmation, but this comparison should not be interpreted as independent biological replication of the association. The Sanger sequencing genotypes were used for subsequent candidate-SNP association analysis.

The association between CM048886.1:g.61357858G>A genotype and reproductive traits was analyzed using the MIXED procedure of SAS 9.4 (SAS Institute Inc., Cary, NC, USA). The model was yijk = μ + Gi + Aj + Pk + eijk, where yijk is the doe-level phenotypic value; μ is the overall mean; Gi is the fixed effect of genotype; Aj is the fixed effect of age at kidding; Pk is the fixed effect of parity; and eijk is the residual error. Least-squares means are presented as mean ± standard error of the mean. For the candidate-SNP association analysis, *p* < 0.05 was considered nominally significant.

## 3. Results

### 3.1. Genome-Wide SNP Distribution and Phenotype Distribution

**Figure 1:**
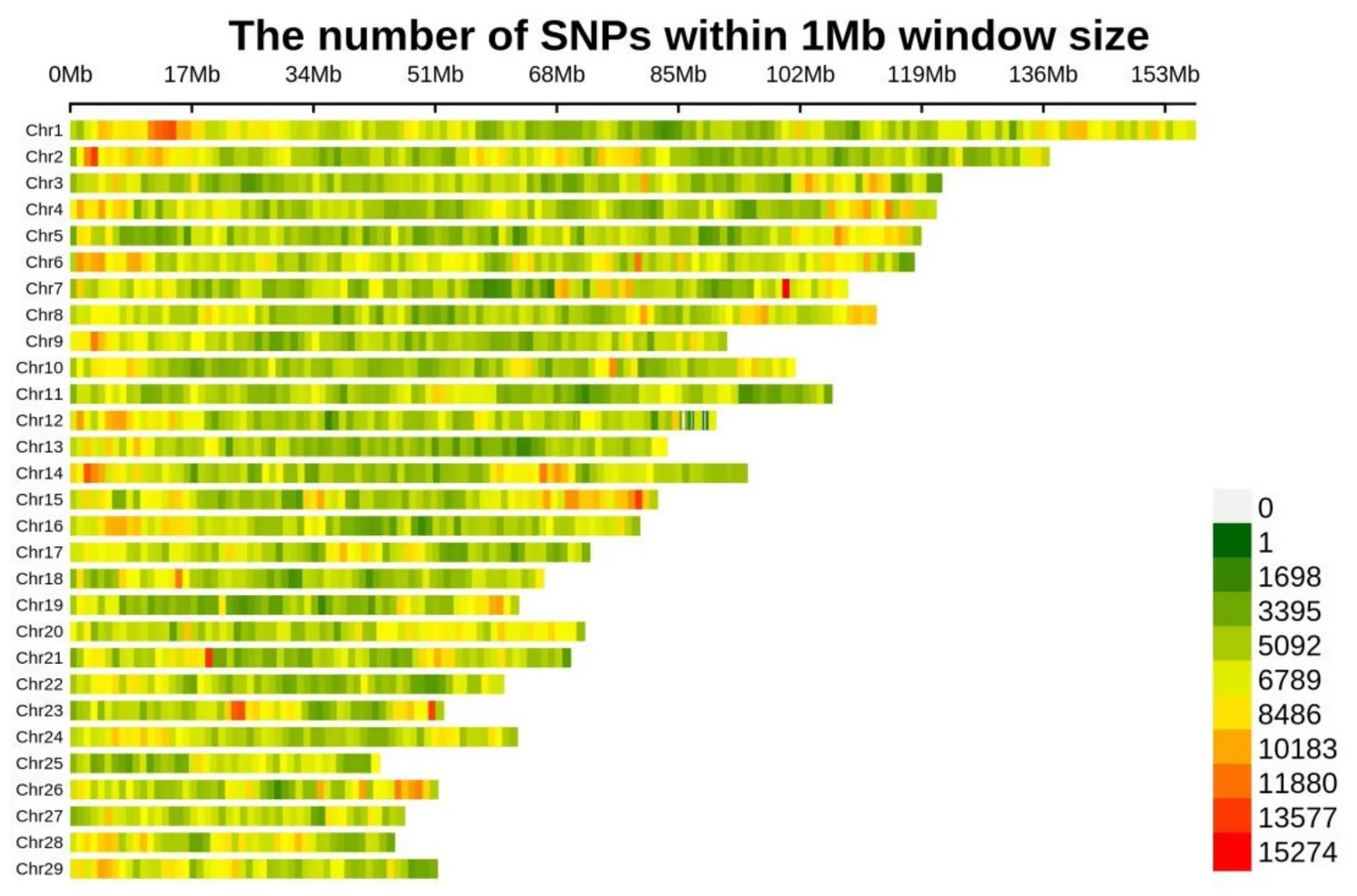
Genome-wide SNP density across goat autosomes using a 1 Mb window size after quality control.

After quality control, 14,597,388 autosomal SNPs were distributed across all 29 autosomes. The 1 Mb window-based density plot showed broad marker coverage of the goat genome, supporting use of the dataset for preliminary association screening (Figure 1).

**Figure 2:**
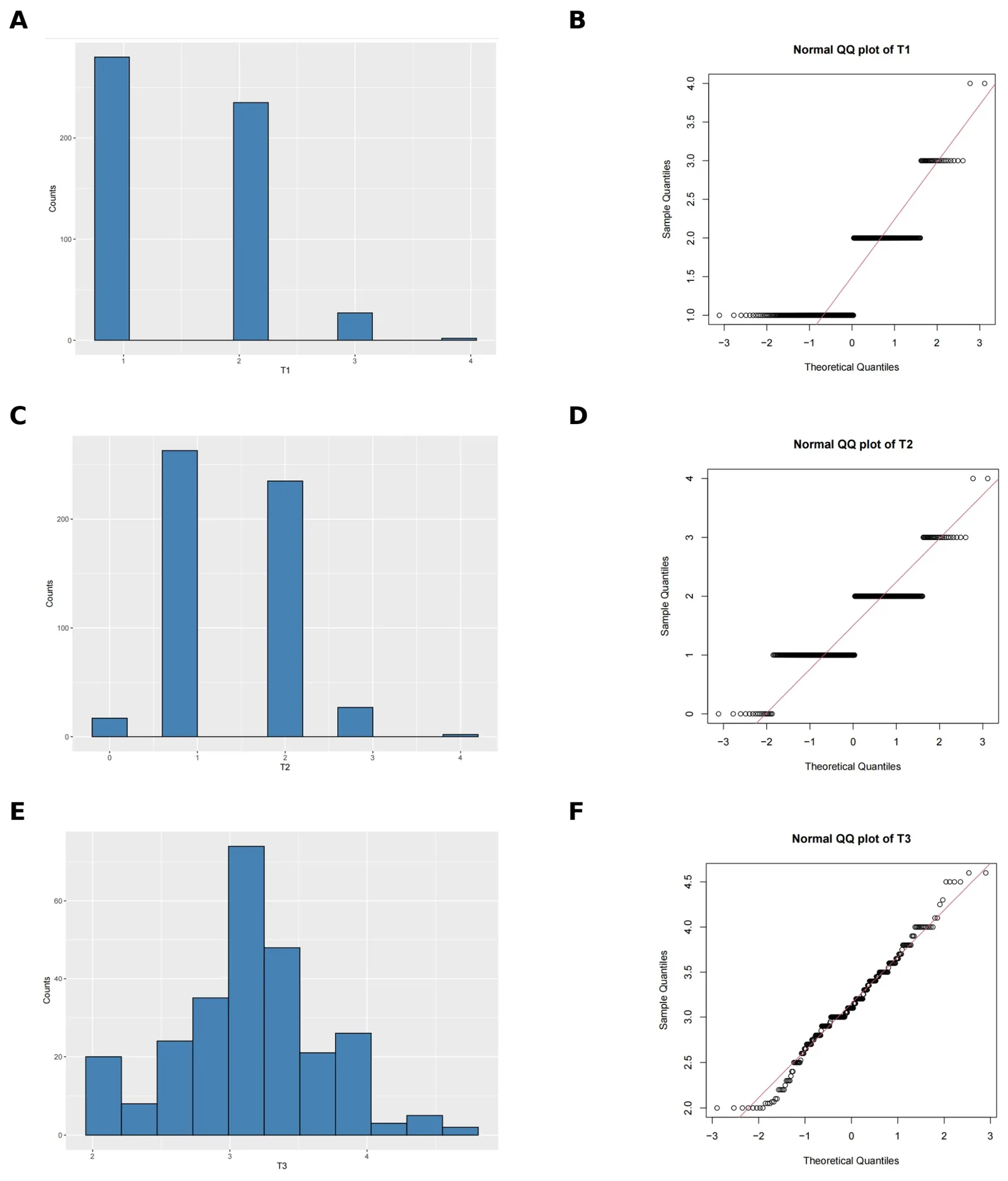
Phenotypic distributions and normal Q-Q plots. (**A**,**B**) Frequency distribution and Q-Q plot for litter size (T1); (**C**,**D**) frequency distribution and Q-Q plot for live-born kid number (T2); (**E**,**F**) frequency distribution and Q-Q plot for average birth weight (T3).

The distributions of the three reproductive traits are shown in Figure 2. Litter size and live-born kid number were discrete count traits concentrated mainly around one and two kids, whereas average birth weight showed a more continuous distribution. The Q-Q plots were inspected to evaluate the distribution of each phenotype before association analysis.

### 3.2. Population Structure and Relatedness

**Figure 3:**
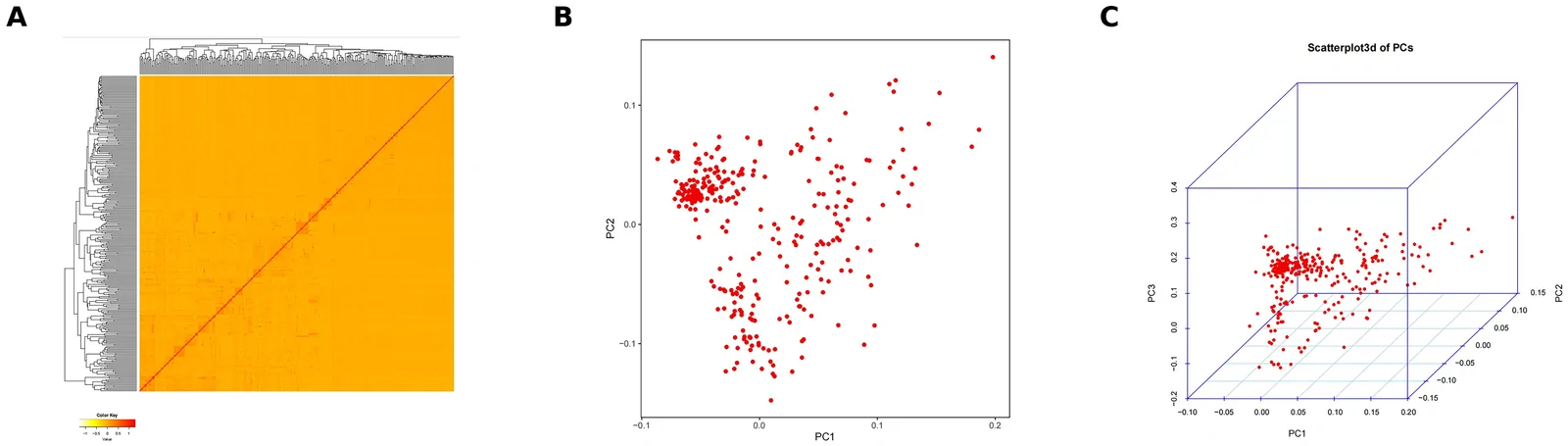
Population structure and relatedness of the Saanen dairy goat population. (**A**) Kinship heatmap; (**B**) two-dimensional PCA plot; (**C**) three-dimensional PCA plot.

The kinship heatmap and PCA plots indicated varying levels of relatedness within the Saanen dairy goat population but did not reveal obvious severe population stratification (Figure 3). This supported the use of a mixed model including a genomic relationship matrix to reduce false positive associations caused by relatedness.

### 3.3. Genome-Wide Association Signals for Reproductive Traits

**Figure 4:**
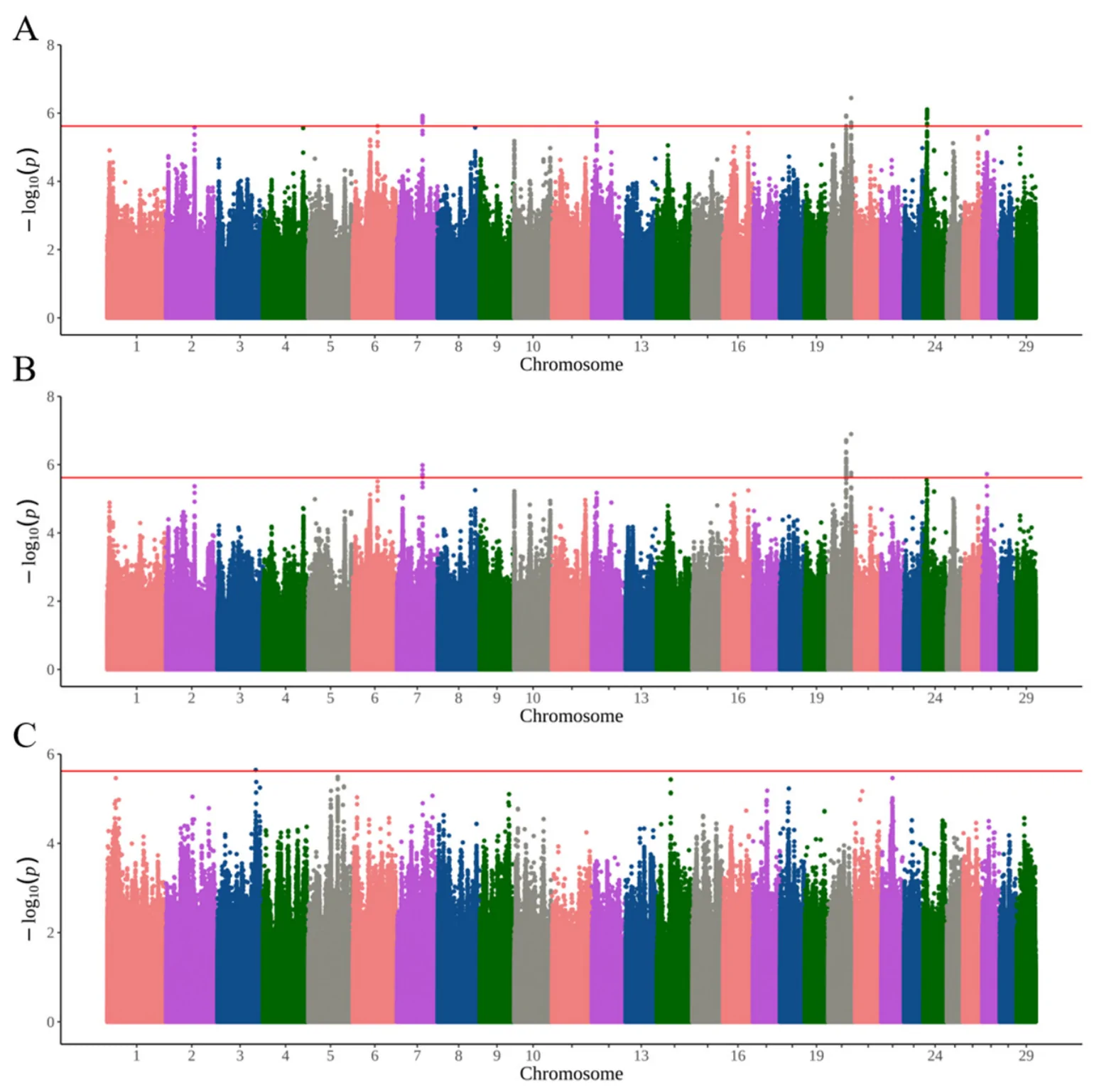
Manhattan plots of genome-wide association analyses for (**A**) litter size, (**B**) live-born kid number, and (**C**) average kid birth weight in Saanen dairy goats. The horizontal red line indicates the genome-wide significance threshold.

Genome-wide association analyses were performed for litter size, live-born kid number, and average kid birth weight (Tables S1–S3), and the corresponding Manhattan plots are presented in Figure 4. Overall, the GWAS results revealed distinct association patterns among the three reproductive traits. For litter size, the strongest association signal was detected on chromosome 20, with the peak SNP located within the candidate interval selected for subsequent analysis. A similar association pattern was observed for live-born kid number, in which the most prominent signal also mapped to the same chromosome 20 region. In contrast, no comparable association signal was detected in this region for average kid birth weight, suggesting that the underlying genetic architecture differed from that of the two reproductive output traits. Because both litter size and live-born kid number shared the same chromosome 20 association signal, this genomic region was prioritized for further investigation. The intergenic SNP CM048886.1:g.61357858G>A, located near *CTNND*2, was subsequently selected for PCR/Sanger genotyping and candidate SNP association analysis.

### 3.4. PCR Amplification and Genotype Confirmation of the Candidate Intergenic SNP Near CTNND2

**Figure 5:**
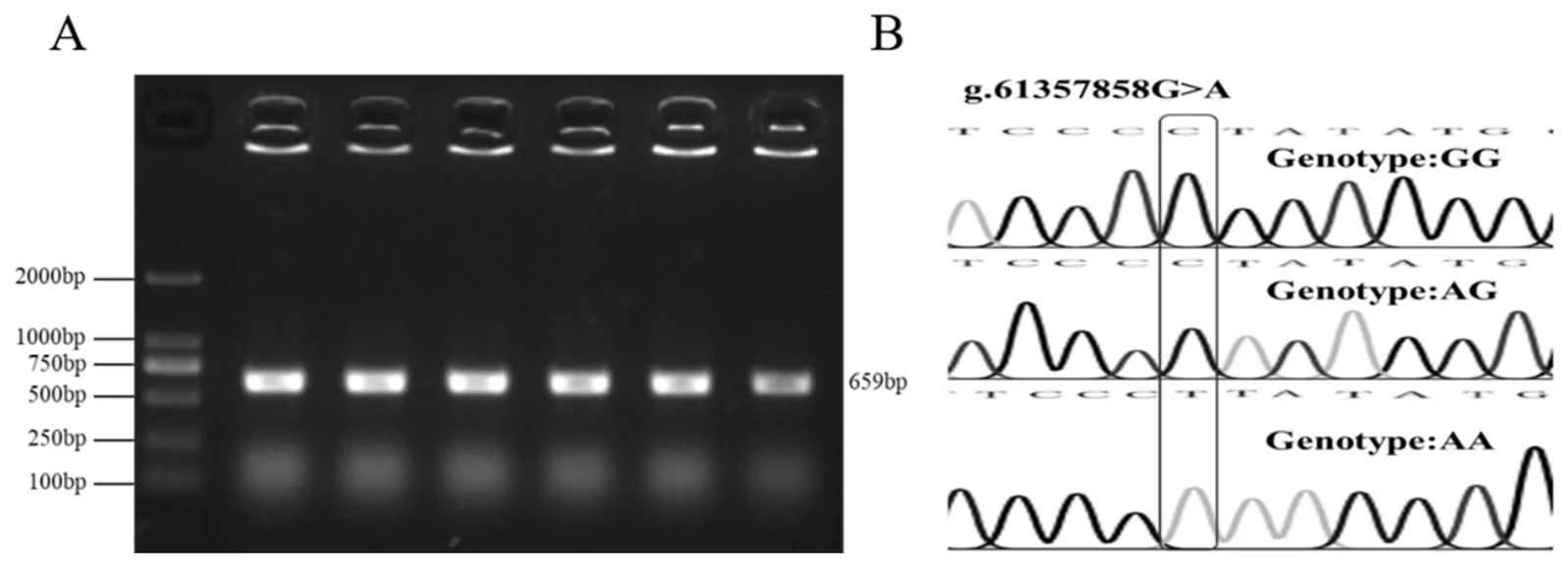
Technical genotype confirmation of CM048886.1:g.61357858G>A, an intergenic SNP near *CTNND2*. (**A**) PCR amplification of the 659 bp target fragment; (**B**) representative Sanger sequencing chromatograms showing GG, AG, and AA genotypes.

The target fragment containing CM048886.1:g.61357858G>A was successfully amplified. A clear band of approximately 659 bp was observed, indicating that the PCR products were suitable for Sanger sequencing (Figure 5A). Sequencing identified the G>A substitution CM048886.1:g.61357858G>A. Three genotypes were detected: GG, AG, and AA (Figure 5B).

### 3.5. Genetic Diversity of CM048886.1:g.61357858G>A

The genotype and allele frequencies and genetic diversity indices are shown in Table 2. The GG genotype was the most frequent genotype (0.569), followed by AG (0.323) and AA (0.109). The G allele was predominant with a frequency of 0.73, whereas the A allele frequency was 0.27. The observed heterozygosity was 0.323, the expected heterozygosity was 0.394, and the PIC value was 0.27, indicating moderate polymorphism. The Hardy–Weinberg chi-square value was 0.001414, suggesting no significant deviation from equilibrium in this population.

### 3.6. Association Between CM048886.1:g.61357858G>A Genotype and Reproductive Traits

**Figure 6:**
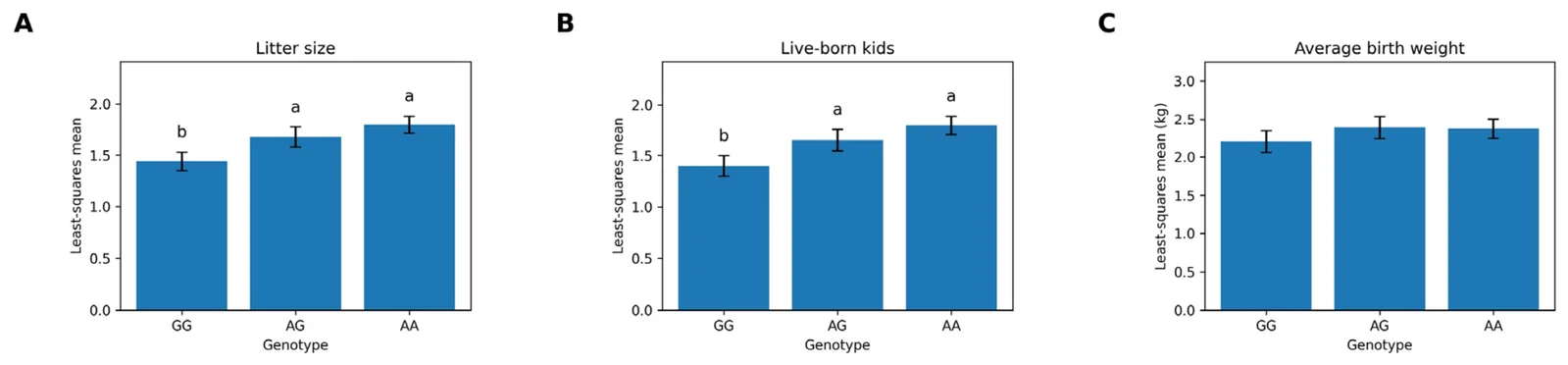
Least-squares (means ± SE) of reproductive traits among CM048886.1:g.61357858G>A genotypes in Saanen dairy goats. (**A**) Litter size; (**B**) live-born kid number; (**C**) average kid birth weight. Different lowercase letters indicate nominal pairwise differences among genotypes (*p* < 0.05).

The associations between CM048886.1:g.61357858G>A genotypes and reproductive traits are summarized in Table 3 and illustrated in Figure 6. Association analysis revealed nominally significant genotype effects on litter size (*p* = 0.0445) and live-born kid number (*p* = 0.0448), whereas no significant effect was detected for average kid birth weight (*p* = 0.125).

For litter size, the least-squares mean increased from the GG genotype (1.438 ± 0.091) to the AG genotype (1.675 ± 0.097) and reached the highest value in the AA genotype (1.796 ± 0.085). No nominal pairwise difference was observed between AG and AA genotypes, whereas both genotypes showed higher litter sizes than the GG genotype in nominal pairwise comparisons (Figure 6A). A similar genotype pattern was observed for live-born kid number, with AG (1.651 ± 0.105) and AA (1.796 ± 0.092) does exhibiting higher values than GG does (1.398 ± 0.099) in nominal pairwise comparisons, while no nominal pairwise difference was detected between AG and AA genotypes (Figure 6B).

In contrast, average kid birth weight did not differ significantly among the three genotypes (*p* = 0.125), although AG and AA animals exhibited numerically higher least-squares means than GG animals (Figure 6C). Overall, AG and AA genotypes were associated with more favorable litter size-related traits than the GG genotype, whereas no apparent genotype effect was observed for average kid birth weight.

## 4. Discussion

The principal finding of this study is the convergence of the genome-wide and candidate-locus analyses on the same chromosome 20 region for two closely related reproductive output traits. Litter size and live-born kid number showed a shared GWAS signal, and the follow-up analysis of CM048886.1:g.61357858G>A in an independent validation population yielded nominal genotype associations with both traits (*p* = 0.0445 and *p* = 0.0448, respectively). Given that the observed *p* values were close to 0.05, these associations should be regarded as nominal and interpreted cautiously until validated in larger populations. The similar direction of the genotype effects across these two phenotypes provides additional consistency: AG and AA animals had higher least-squares means than GG animals for both traits. By contrast, the absence of an association with average kid birth weight suggests that the observed effect may be more closely related to reproductive output than to neonatal growth. Importantly, the association of CM048886.1:g.61357858G>A with litter size and live-born kid number in an independent validation population provides additional support for the chromosome 20 GWAS signal and strengthens the evidence that this genomic region may contribute to variation in reproductive performance in Saanen dairy goats.

The location of this signal is also informative in the context of previous goat studies. GWAS in Markhoz, Dazu Black, and Youzhou Dark goats have implicated different genomic regions and genes in litter-size variation [3,7,8], while candidate-gene studies have reported associations involving *KISS*1, *GDF*9, *BMPR*1*B*, and *BMP*15 [22,23,24,25,26]. The chromosome 20 signal observed here therefore adds a distinct Saanen-specific result to a literature characterized by considerable locus heterogeneity. Rather than indicating inconsistency among studies, this pattern is compatible with differences in allele frequency, linkage disequilibrium, genetic background, management, and phenotype definition among goat populations. It also emphasizes that marker effects identified in one breed should not be assumed to transfer directly to another without population-specific validation.

CM048886.1:g.61357858G>A is intergenic and lies approximately 552,627 bp from *CTNND*2. *CTNND*2 remains biologically plausible because delta-catenin participates in cadherin-associated cellular processes and Wnt/beta-catenin signaling, and WNT signaling is relevant to ovarian follicular development and granulosa-cell function [9,10,11,12,13]. Nevertheless, physical proximity alone is insufficient to assign causality. The detected SNP may itself have a regulatory effect, may be in linkage disequilibrium with another functional variant, or may simply mark a broader associated haplotype. Accordingly, the present data support prioritization of this chromosome 20 interval, but they do not establish *CTNND*2 as the causal gene or CM048886.1:g.61357858G>A as the causal mutation.

The population-genetic and phenotypic results further define the potential value and current limits of this locus. The SNP showed moderate polymorphism (PIC = 0.27) and no departure from Hardy–Weinberg equilibrium, indicating that both alleles segregate in the studied population. Practically, this moderate PIC indicates useful but not high allelic informativeness; therefore, the locus may contribute to marker-assisted selection only after validation and preferably as part of a multi-marker strategy rather than as a stand-alone selection criterion. The favorable direction of the A allele was consistent for litter size and live-born kid number, but the AA class was relatively small (n = 33), which limits statistical power and the precision of the AA genotype effect estimate, and the observed associations were nominal. Thus, CM048886.1:g.61357858G>A is better regarded at this stage as a candidate marker for further testing than as a marker ready for routine selection. Before breeding application, its effect size and predictive value should be evaluated in larger Saanen populations, across herds and parities, and together with potentially correlated traits such as kid survival, doe health, and milk production.

The main remaining questions concern reproducibility and biological mechanism. Independent replication is needed to determine whether the chromosome 20 association persists beyond this single herd and to obtain more precise estimates of the genotype effect. Dense fine-mapping and haplotype analysis could determine whether CM048886.1:g.61357858G>A tracks a narrower functional interval, while transcriptomic or regulatory analyses of reproductive tissues could test whether variation in this region is associated with *CTNND*2 or other nearby genes. Functional assays would then be required to distinguish a causal regulatory variant from a linked marker. Addressing these steps would convert the present association signal from a promising population-level observation into evidence that can be evaluated reliably for marker-assisted or genomic breeding.

## 5. Conclusions

This study identified a chromosome 20 genomic region associated with litter size-related traits in Saanen dairy goats through genome-wide association analysis and candidate SNP validation. The intergenic variant CM048886.1:g.61357858G>A, located near *CTNND*2, was associated with litter size and live-born kid number but not with average kid birth weight, suggesting that this genomic region may contribute specifically to reproductive performance. Although the causal gene and molecular mechanism remain to be elucidated, the present study expands current knowledge of the genetic architecture underlying litter size in dairy goats and identifies a promising candidate marker for future validation and molecular breeding aimed at improving reproductive efficiency.

## Figures and Tables

**Table 1:** Primer information for amplification of CM048886.1:g.61357858G>A, an intergenic SNP near *CTNND2*.

Primer	Sequence (5′–3′)	Amplicon Size (bp)	Target SNP
Forward	GGGACTTAGTGTATAGTACAGGGA	659	CM048886.1:g.61357858G>A
Reverse	ACACAGTGAATCACAGCGCA	659	CM048886.1:g.61357858G>A

**Table 2:** Genetic parameters of CM048886.1:g.61357858G>A in Saanen dairy goats.

Parameter	Value
Locus	CM048886.1:g.61357858G>A
Allele frequency of G	0.73
Allele frequency of A	0.27
GG genotype	173 (0.569)
AG genotype	98 (0.322)
AA genotype	33 (0.109)
Observed heterozygosity (Ho)	0.323
Expected heterozygosity (He)	0.394
Effective allele number (Ne)	1.65
Polymorphism information content (PIC)	0.27
Hardy–Weinberg χ^2^	0.001414

**Table 3:** Association between CM048886.1:g.61357858G>A and reproductive traits in Saanen dairy goats.

Locus	Genotype	*n*	Litter Size	Live-Born Kids	Average Birth Weight (kg)
CM048886.1:g.61357858G>A	GG	173	1.438 ± 0.091	1.398 ± 0.099	2.204 ± 0.142
	AG	98	1.675 ± 0.097	1.651 ± 0.105	2.387 ± 0.150
	AA	33	1.796 ± 0.085	1.796 ± 0.092	2.372 ± 0.129
	P		0.0445	0.0448	0.125

## Data Availability

The original contributions presented in this study are included in the article/Supplementary Materials. Further inquiries can be directed to the corresponding author.

## Supplementary Materials

The following supporting information can be downloaded at: https://www.mdpi.com/article/10.3390/vetsci13090961/s1, Table S1: Genome-wide association study (GWAS) results for litter size (T1) in Saanen dairy goats; Table S2: Genome-wide association study (GWAS) results for live-born kid number (T2) in Saanen dairy goats; Table S3: Genome-wide association study (GWAS) results for average kid birth weight (T3) in Saanen dairy goats.
